# On a Continuous Aqueous Thermogalvanic Redox Agent with Anomalous Thermopower

**DOI:** 10.1021/acs.nanolett.5c02774

**Published:** 2025-07-24

**Authors:** Ehsan Hosseini, Mohammad Zakertabrizi, Mina Hosseini, Matthew. J. Powell-Palm

**Affiliations:** a J. Mike Walker ’66 Department of Mechanical Engineering, 2655 Texas A&M University, College Station, Texas 77803, United States; b Department of Materials Science and Engineering, 2655 Texas A&M University, College Station, Texas 77803, United States; c Department of Biomedical Engineering, 2655 Texas A&M University, College Station, Texas 77803, United States

**Keywords:** Low-grade heat, thermogalvanic, energy harvesting, thermoelectric, Seebeck effect

## Abstract

Achieving stable and sustained power output remains a major challenge in the development of ionic thermoelectric devices (such as thermogalvanic cells and thermoionic capacitors) for low-grade heat harvesting. Many such systems exhibit strong performance in the initial throes of operation but degrade rapidly over time, limiting their utility. Here, we report an aqueous thermogalvanic redox agent ([Ni­(bpy)_3_]^2+/3+^) with a Seebeck coefficient approximately double that of the canonical [Fe­(CN)_6_]^4‑/3–^, which can be utilized continuously for multiple hours under large temperature gradients (Δ*T* ≥ 60 K) without significant change in electrical performance. Molecular dynamics simulations suggest that significant differences in hydration shell behavior between the oxidant and reductant, which in turn provide a significant (here configurational) entropy difference, drive the observed thermopower. This new redox pair demonstrates stability, cyclability, and tunability in both liquid- and gel-based electrolytes, and provides a novel redox platform from which to build next-generation high-thermopower waste-heat recovery cells.

## Graphical abstract

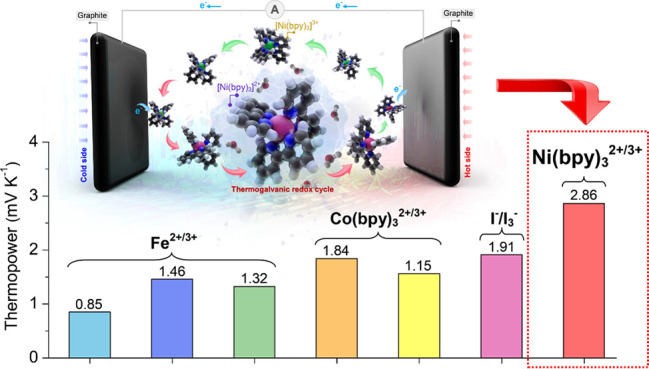

Efficient recovery of low-grade waste heat (that generated at temperatures below 100 °C) remains a persistent challenge in energy science. Despite representing over half of the ∼80 PWh of global waste heat generated annually, this resource remains largely untapped due to limitations in thermal energy conversion technologies.^1–3^ Thermoelectric energy harvesting, which broadly speaking employs the Seebeck effect to directly convert temperature gradients into electrical power, offers a long-sought pathway to reclaim this energy. However, conventional solid-state electronic thermoelectric (e-TE) materials typically exhibit low Seebeck coefficients (on the order of μV K^–1^), acutely restricting their utility at sub-100 °C temperatures.^4,5^

Ionic thermoelectric (i-TE) systems (including thermo-ionic capacitors (TICs) and thermogalvanic cells (TGCs)) have recently emerged as promising alternatives, exhibiting Seebeck coefficients in the millivolt-per-kelvin (mV K^–1^) range.^6–8^ TICs operate on the principle of ionic thermodiffusion, which drives charge separation at the electrode–electrolyte interface and enables high (if ephemeral) thermopower (order 1–10 mV K^–1^), but results in poor continuity of performance and limited electrical power output due to slow charge/discharge kinetics.^8^ TGCs, by contrast, rely on temperature-dependent shifts in redox potential to drive continuous energy generation and typically offer higher electrical power output^3,9–11^ and longevity of operation, but remain hampered by lower thermopower (0.1–3 mV K^–1^).^12^

Despite this challenge, aqueous TGCs have attracted growing interest due to their inherent safety, scalability, and sustained performance under prolonged operation, and the state-of-the-art has advanced considerably in recent years.^12–14^ Over the past decade in particular, this advance has been powered by aqueous cell chemistries that are overwhelmingly based upon the canonical ­[Fe­(CN)_6_]^4‑/3‑^ redox couple.^9,15^ While many strategies have been employed to increase the output and efficiency of cells based on this couple, incorporating complementary ionic diffusion effects, manipulating physical aspects of the redox environment, employing gelation and phase change, etc., few alternative redox couples have been identified with significantly superior continuous redox thermopower.^1,16,17^ Furthermore, despite the 100 °C limit typically ascribed to low-grade heat harvesting,^18^ the operating windows of many thermogalvanic cells are limited to temperature gradients Δ*T* < 20 K.^19–23^ Though select works have reported thermogalvanic heat harvesting from larger gradients,^9,24,25^ gradients Δ*T* ≳ 50 K are often accompanied by challenges such electrolyte instability and reduced Seebeck coefficients.^8,26,27^ Maximizing operating voltage thus remains a key challenge in these systems, with individual cells struggling to exceed 100 mV^27,28^ and the select few that do often suffering from single μW power generation.^1,12,29^

Recent work has suggested a variety of strategies to overcome these limitations and enhance the performance of purely redox-driven thermogalvanic systems, including the incorporation of ionic additives to regulate redox ion distribution, the use of solid-to-liquid phase transitions to amplify entropy changes and thermopower, and the development of hybrid systems that synergistically combine multiple functional components to improve voltage output and stability under larger temperature gradients.^3,30–32^ Notable examples include the work of Zhou et al.,^33^ which showed that α-cyclodextrin enables cold-side encapsulation of I_3_^–^, increasing thermopower from −0.86 to −1.97 mV K^–1^, and the work of Yu et al.,^9^ which introduced guanidinium ions to induce selective crystallization of Fe­(CN)_6_^4–^ at the cold electrode, with gravity-driven transport to the hot side, which achieved a high p-type thermopower of 3.7 mV K^–1^.

In this work, we report a novel self-sustaining thermogalvanic redox couple based on tris­(2,2′-bipyridine)­nickel­(II)/(III), abbreviated [Ni­(bpy)_3_]^2+/3+^, which possesses roughly double the Seebeck coefficient of [Fe­(CN)_6_]^4‑/3–^ in both pure water and polyvinyl alcohol (PVA)-based hydrogels, produces anomalously large continuous voltage (up to ∼180 mV) absent additional molecular additives, and remains operable over a wide temperature delta (Δ*T* ≥ 60 K) without diminishing Seebeck. We probe the entropic origins of this thermopower via molecular simulation, characterize the performance of simple water- and hydrogel-[Ni­(bpy)_3_]^2+/3+^ thermogalvanic cells, and demonstrate that generic modifications of the aqueous electrolyte (enhancement of electrical conductivity, frustration of bulk water structure, etc.) can dramatically further increase the cells’ power and current density. Our results suggest that the [Ni­(bpy)_3_]^2+/3+^ couple may provide a powerful redox foundation for versatile, high-thermopower thermogalvanic cells.

Our [Ni­(bpy)_3_]^2+/3+^ thermogalvanic cell, depicted in Figure 1a, employs a planar structure with two graphite electrodes in a [Ni­(bpy)_3_]^2+/3+^ electrolyte for continuous operation. When a temperature gradient is applied across the cell, the [Ni­(bpy)_3_]^2+/3+^ redox couple engages in a reversible reaction, producing a voltage difference between the electrodes. This thermally driven redox process sustains charge movement between the hot and the cold sides, converting the temperature difference into electrical energy. Specifically, on the hot side, [Ni­(bpy)_3_]^2+^ oxidizes to [Ni­(bpy)_3_]^3+^ by donating an electron to the electrode, while on the cold side, [Ni­(bpy)_3_]^3+^ reduces to [Ni­(bpy)_3_]^2+^ by accepting an electron from the electrode. In Figure 1b, we probe the thermopower performance as a function of [Ni­(bpy)_3_]­Br_2_ concentration (ranging from 0.01 M to 0.05 M, the approximate solubility limit in water) in both pure water and hydrogel (PVA) substrates. A significant increase in thermopower is observed near 0.04 M, with values reaching approximately 2.14 mV K^–1^ in the liquid phase and 2.86 mV K^–1^ in the hydrogel phase at 0.05 M.

**Figure 1:**
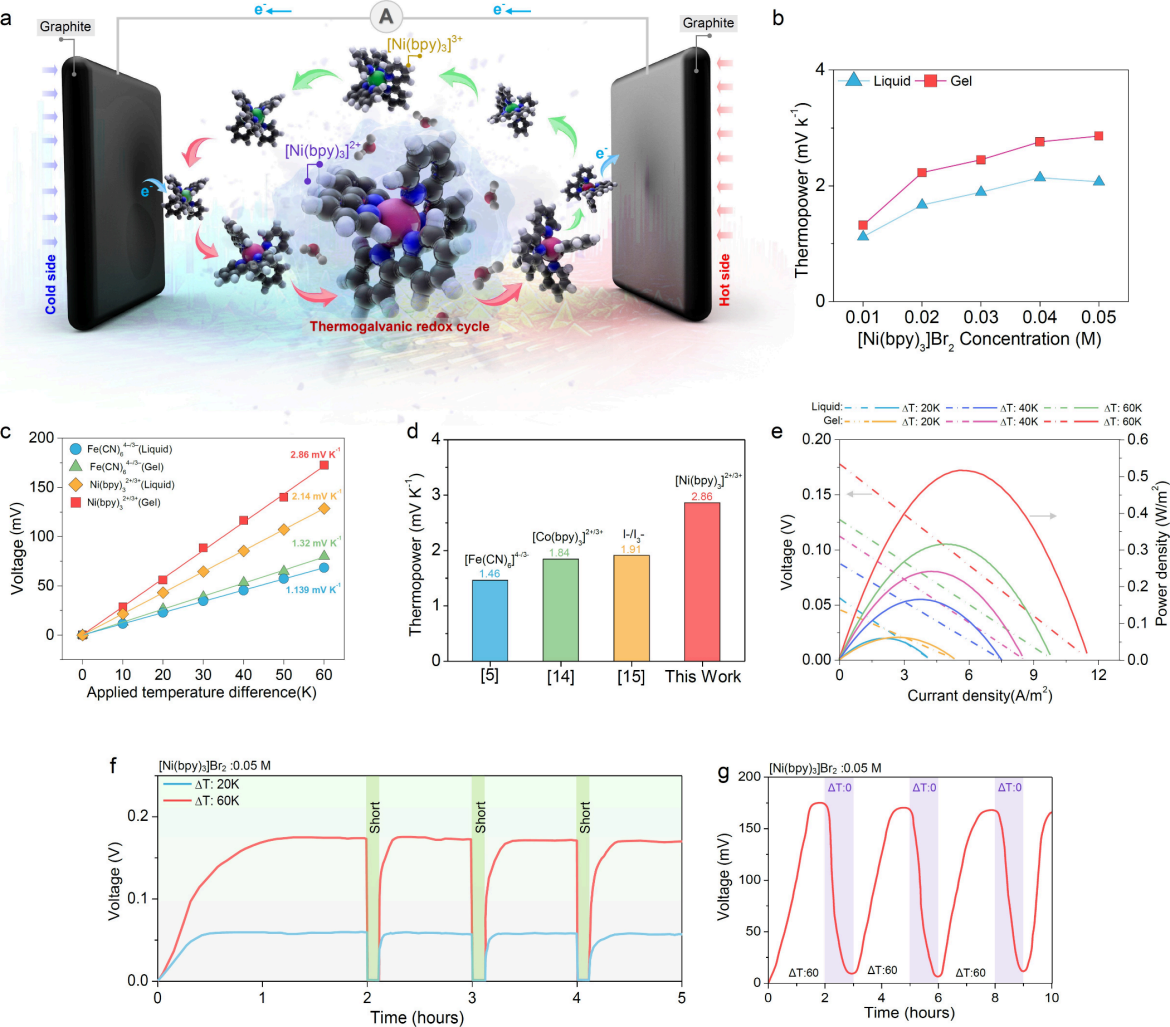
Characterization of thermogalvanic cells based on the [Ni­(bpy)_3_]^2+/3+^ redox couple. (a) Schematic representation of thermogalvanic [Ni­(bpy)­3]^2+/3+^ redox couple under a temperature gradient. (b) Thermopower of liquid and gel electrolytes as a function of [Ni­(bpy)_3_]­Br_2_ concentration. (c) Cell potential and thermopower of [Ni­(bpy)_3_]^2+/3+^ and [Fe­(CN)_6_]^4‑/3–^ in liquid and PVA hydrogel phases over Δ*T* range 0–60 K with cold side electrodes at 283 K and hot side electrodes at 283 K + Δ*T*. (d) Thermopower comparison between [Ni­(bpy)_3_]^2+/3+^ to [Fe­(CN)_6_]^4‑/3–^, [Co­(bpy)_3_]^2+/3+^, and I^–^/I_3_^–^ redox couples. Literature values shown reflect the maximum continuous redox thermopower reported in water or gel-based cells.^16,37,38^ (e) Voltage (dashed lines, left *y*-axis), current, and power (solid lines, right *y*-axis) density curves of [Ni­(bpy)­3]^2+/3+^ in water and PVA hydrogel at Δ*T* of 20, 40, and 60 K, using graphite electrodes in a 10 mm × 10 mm × 35 mm cell. (f) Voltage stability of [Ni­(bpy)_3_]^2+/3+^ at 0.05 M concentration under Δ*T* of 60 K over 5 h, with electrical discharge via short circuit, showing consistent peak thermopower across multiple cycles. (g) Voltage cyclability of [Ni­(bpy)_3_]^2+/3+^ (0.05 M) at Δ*T* of 60 K across thermal discharge cycles caused by removing temperature gradient.

To provide a better understanding of the advantages of [Ni­(bpy)_3_]^2+/3+^, we conducted comparative tests using [Fe­(CN)_6_]^4–/3–^, a well-established and widely recognized thermogalvanic material, within our experimental setup. In Figure 1.c, we show paired cell potential and thermopower results for [Ni­(bpy)_3_]^2+/3+^ and [Fe­(CN)_6_]^4–/3–^ in water and hydrogel configurations, evaluated over a Δ*T* range from 0 to 60 K (experimental details available in Methods section and Supplementary Note 1. The [Fe­(CN)_6_]^4–/3–^ thermopower measured here aligns well with prior literature^9,15^ ). Cell potentials scale linearly with the Δ*T* in this temperature window, and thermopowers are derived from their slopes.

In both liquid and hydrogel cells, [Ni­(bpy)_3_]^2+/3+^ shows dramatically superior thermopower compared to [Fe­(CN)_6_]^4–/3–^ (Figure 1c), and it furthermore outperforms several other redox couples reported in the literature (Figure 1d). In Figure 1e, we show corresponding voltage–current density–power density curves for these cells, which increase predictably with Δ*T* (Figure 1c and Figures S1–S3).

We attribute the increased thermopower and power density of the hydrogel form (2.86 mV K^–1^) compared to the liquid form (2.14 mV/K) to interrelated effects variously reported in the literature: interference in both bulk water and hydration shell structures, which can enhance the solvation entropy differences between redox species (discussed further below); further modification to ion hydration shells driven by polymer–ion interactions that do the same; and the hydrogel’s reduced thermal conductivity, which reduces conduction losses and helps to maintain a steeper temperature gradient, thereby amplifying voltage.^20,31,34,35^

In Figure 1f,g, we demonstrate the continuous redox of thermogalvanic [Ni­(bpy)_3_]^2+/3+^ cells, which maintain a stable thermal gradient-dependent reduction–oxidation cycle (between [Ni­(bpy)_3_]^2+^ and [Ni­(bpy)_3_]^3+^)^36^ over exceptional periods of operation. Figure 1f shows the remarkable voltage stability of the [Ni­(bpy)_3_]^2+/3+^ pair under a large temperature gradient (Δ*T* = 20 and 60 K), rapid recovery of thermopower generation following electrical discharge (via short circuit), and consistent peak thermopower over multiple hour-long cycles, retaining almost full initial performance. Figure 1g highlights the analogous cyclability and stability upon thermal discharge (via the removal of the temperature gradient).

To further confirm the longevity of these cells, we stored the aqueous ([Ni­(bpy)_3_]­Br_2_) cell used to generate the data in Figure 1f for 20 days, then repeated this charge/discharge experiment (Figure S4). The resulting voltage profile remarkably replicates that shown in Figure 1f, underscoring the chemical stability of the cell and the robustness of the thermogalvanic redox cycle, which operates independent of transient effects (e.g., ionic diffusion or irreversible (i.e., one-way) reactions) that might only temporarily enhance thermopower and/or necessitate periodic recharging for sustained operation.^1,6^

In Figure 1c, we observed that the thermopower of [Ni­(bpy)_3_]^2+/3+^ is significantly stronger than that of [Fe­(CN)_6_]^4‑/3–^. To interrogate the mechanism driving this difference, and the exceptional thermopower of [Ni­(bpy)_3_]^2+/3+^ more generally, we performed paired molecular dynamics simulations of both [Ni­(bpy)_3_]^2+/3+^ and [Fe­(CN)_6_]^4–/3–^ in water (Figure 2a–e, Figure S8). Figure 2a and Figure 2b depict [Ni­(bpy)_3_]^2+^ and [Ni­(bpy)_3_]^3+^, respectively, each coordinated by three bipyridine ligands, surrounded by water molecules forming first hydration shells at approximately 4.56 and 4.21 Å for [Ni­(bpy)_3_]^2+^ and [Ni­(bpy)_3_]^3+^, respectively.

**Figure 2:**
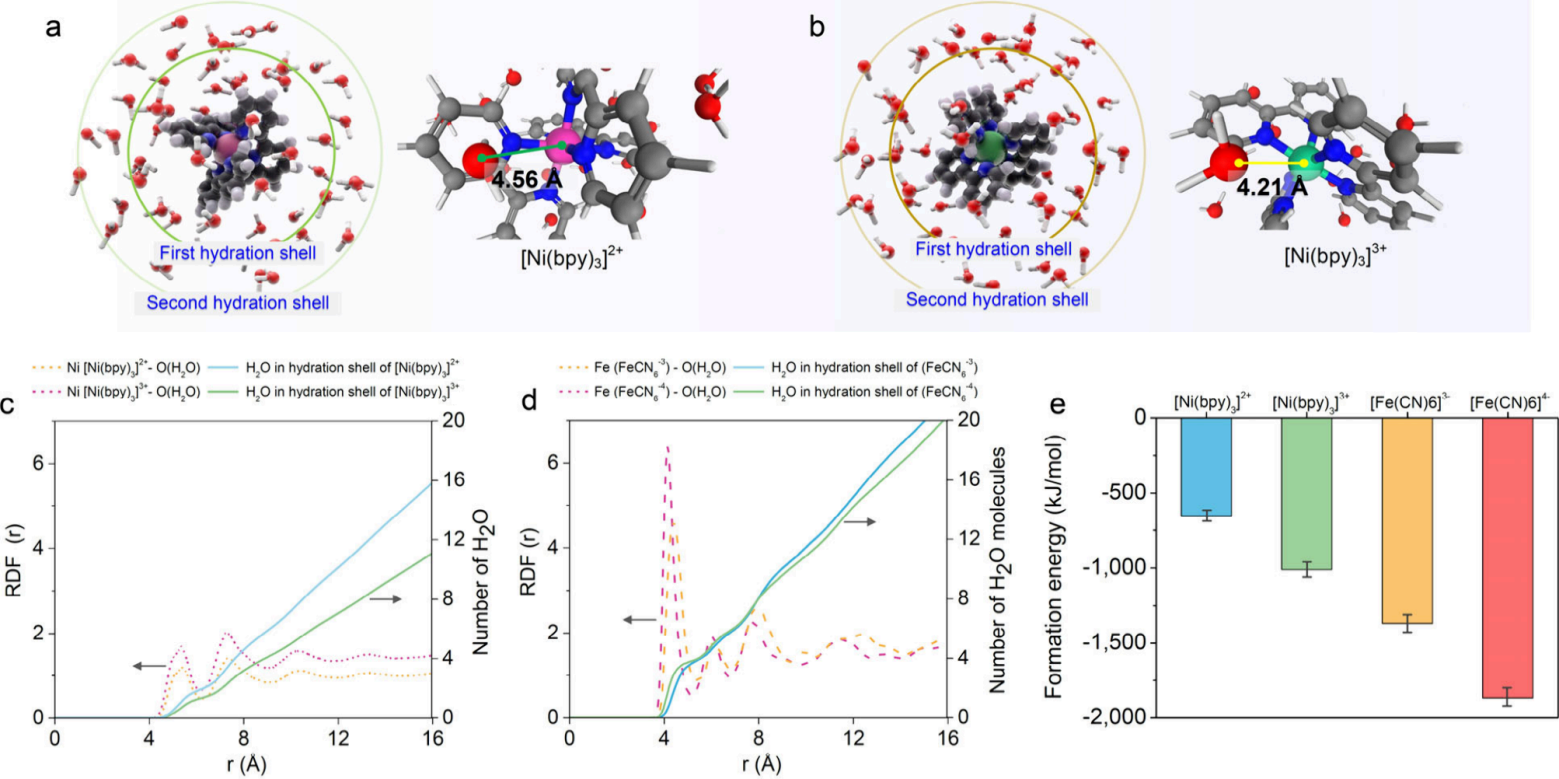
Characterization of thermogalvanic cells based on the [Ni­(bpy)_3_]^2+/3+^ redox couple. (a) Molecular structure of [Ni­(bpy)_3_]^2+^, illustrating the spatial arrangement of the first and second hydration shells surrounding the complex and highlighting water molecule interactions. (b) Molecular structure of [Ni­(bpy)_3_]^3+^, illustrating the spatial arrangement of the first and second hydration shells surrounding the complex and highlighting water molecule interactions. (c) Radial distribution function (RDF) of Ni from [Ni­(bpy)_3_]^2+/3+^ and O from water (left *y*-axis) and number of H_2_O molecules in the coordination shell (right *y*-axis). (d) Radial distribution function (RDF) of Fe from [Fe­(CN)_6_]^4–/3–^ and O from water (left *y*-axis) and the number of H_2_O molecules in the coordination shell (right *y*-axis). (e) Formation energy between H_2_O molecules with the reductant and oxidant ions in [Ni­(bpy)_3_]^2+/3+^ and [Fe­(CN)_6_]^4–/3–^. Bar plots give average values, and error bars give standard deviation, calculated from an aggregated pool of at least 15 distinct molecules for each species, gathered across 3 technical repeat simulations. All simulation details provided in Supplementary Note 1.

We identified that compared to [Fe­(CN)_6_]^4–/3–^, the oxidant and reductant in [Ni­(bpy)_3_]^2+/3+^ both have relatively weak hydration shells, as indicated by reduced water coordination in the radial distribution functions shown in Figure 2c,d and the reduced (less negative) formation energies shown in Figure 2e. Furthermore, the number of water molecules involved in these shells differs significantly between the 2+ and 3+ states, with [Ni­(bpy)_3_]^2+^ containing ∼45% more water within a 16 Å distance than [Ni­(bpy)_3_]^3+^. We hypothesize that this configurational difference between redox species may contribute significantly to the characteristic entropy difference required for thermopower,^1,15^ and we note that the hydration shells of [Fe­(CN)_6_]^4–/3–^ are considerably less different from one another (Figure 2d), plausibly consistent with our observation of higher thermopower in [Ni­(bpy)_3_]^2+/3+^.

We further hypothesize that the combination of relatively weak hydration shells and substantial differences in hydration behaviors between redox states may render the thermogalvanic redox process of [Ni­(bpy)_3_]^2+/3+^ more sensitive to entropic changes in the surrounding hydrogen bond network (such as those imparted by the addition of PVA), which may differentially affect one redox state more than the other according to the hydration shell strength, thereby further increasing the entropy difference driving thermopower. This observation provides one explanation for the relatively stronger impact of PVA on the [Ni­(bpy)_3_]^2+/3+^ thermopower observed in Figure 1c and suggests that the [Ni­(bpy)_3_]^2+/3+^ system may be broadly more susceptible to thermopower enhancement via simple entropic manipulation of the surrounding solvent. Future work may seek to more deeply interrogate this putative link between oxidant and reductant solvation shell configuration and the resulting thermopower across a variety of thermogalvanic redox species.

In addition to the continuous nature of its thermogalvanic redox cycle and its comparatively wide window of temperature operation, [Fe­(CN)_6_]^4–/3–^ has remained the canonical thermogalvanic redox agent because of the broad tunability of its performance, which lends itself to rich hybridization and enhancement across many cell chemistries.^6,15^ We next demonstrate similarly straightforward performance enhancements in a hydrogel [Ni­(bpy)_3_]^2+/3+^ cell, wherein generic augmentation of the charge carrier rate in the electrolyte and enhancement of electrode surface characteristics markedly improve current and power output.

To increase the charge carrier rate, we added varying concentrations of NaBr to our [Ni­(bpy)_3_]^2+/3+^ hydrogel electrolyte (Figure 3a), with current densities observed to increase with a NaBr content up to 0.5 M (the maximum concentration tested). Across operating temperatures, the addition 0.5 M NaBr provided a nearly 3-fold increase in power density, attributed to enhanced electrical conductivity (Figure S5). This addition was also observed to significantly increase the energy density, with peak densities of 0.75 kJ m^–2^ at 42 Ω (Δ*T* = 20 K), 2.63 kJ m^–2^ at 42 Ω (Δ*T* = 40 K), and 5.94 kJ m^–2^ at 57 Ω (Δ*T* = 60 K) (Figure 3b). These results indicate superior electrochemical performance and enhanced charge transfer at higher temperatures and the potential simplicity of additional performance enhancement in [Ni­(bpy)_3_]^2+/3+^ cells.

**Figure 3:**
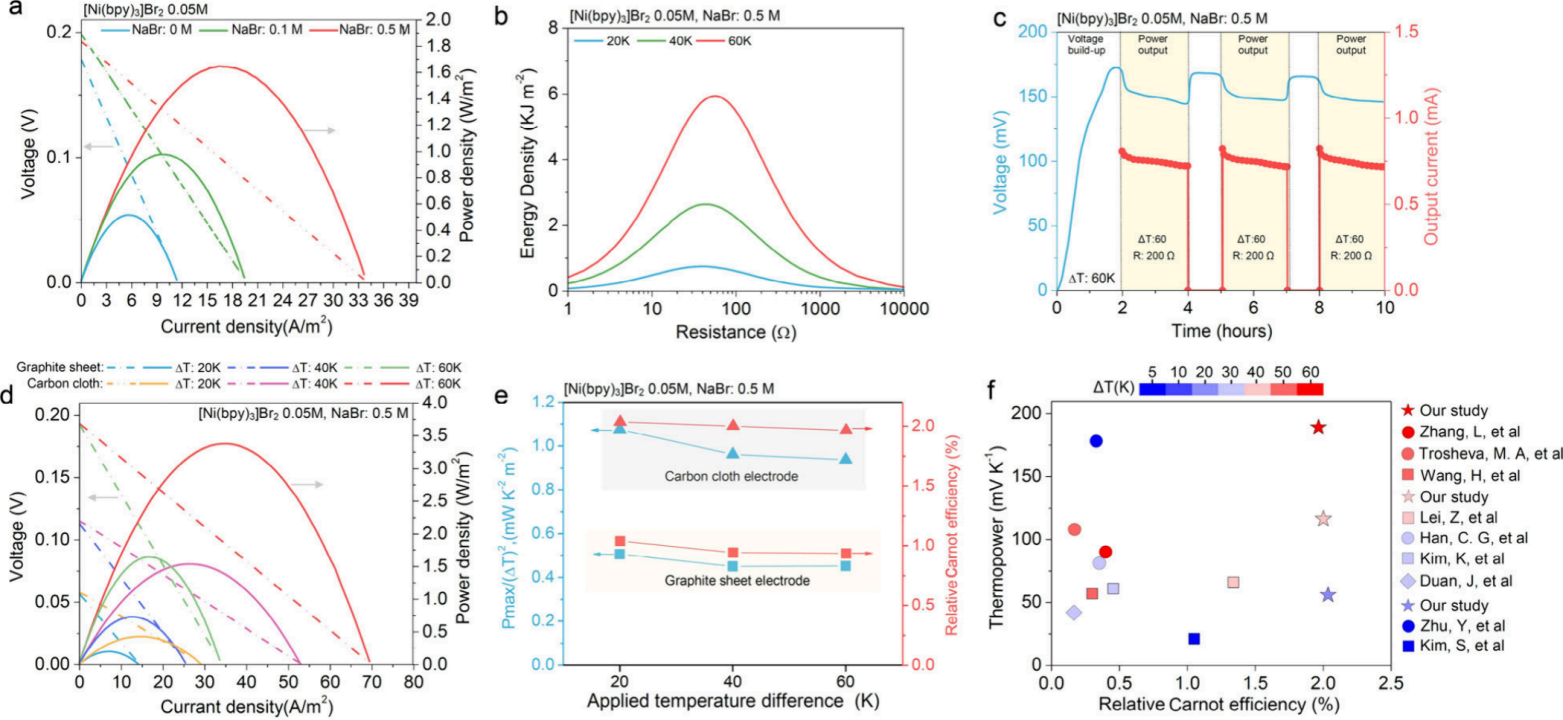
Tunability of thermogalvanic performance in [Ni­(bpy)_3_]^2+/3+^ cells. (a) Voltage and power density versus current density for a gel electrolyte at Δ*T* = 60 K with NaBr concentrations of 0 M, 0.1 M, and 0.5 M. (b) Energy density vs resistance for [Ni­(bpy)_3_]­Br_2_ 0.05 M, NaBr 0.5 M at 20 K, 40 K, and 60 K. (c) Voltage and current output stability of [Ni­(bpy)_3_]­Br_2_ at 0.05 M with NaBr at 0.5 M under Δ*T* of 60 K over 10 h, with voltage and current output measured at 200 Ω load. (d) Voltage, current density, and power density curves of [Ni­(bpy)_3_]­Br_2_ at 0.05 M with NaBr at 0.5 M in hydrogel with different carbon-based electrode structures at Δ*T* of 20, 40, and 60 K. (e) Maximum normalized power density and relative Carnot efficiency of [Ni­(bpy)_3_]­Br_2_ at 0.05 M with NaBr at 0.5 M across Δ*T* of 20 K, 40 K, and 60 K with graphite sheet electrodes and carbon cloth electrodes. (f) Assessment of the relative Carnot efficiency and voltage of the [Ni­(bpy)_3_]^2+/3+^ hydrogel system ([Ni­(bpy)_3_]­Br_2_, 0.05 M; NaBr, 0.5 M with carbon cloth electrodes) over temperature differences of 20 K, 40 K, and 60 K, in comparison to other purely redox thermogalvanic materials in liquid and gel phases as reported in recent literature.^3,15,23,25,29,41–45^

Another key strength of thermogalvanic cells (as distinct from thermoionic capacitors) lies in their ability to provide sustained power output. As such, in Figure 3c, we assessed the voltage and current stability of our NaBr enhanced cell over extended periods. Under a 200 Ω load and Δ*T* = 60 K, outputs remained stable across multiple 2 h discharge cycles, with only a ∼3% (∼0.02 mA) drop in current observed during a given discharge. To further demonstrate the continuous redox nature of our cell’s output, we also conducted a 20 h continuous discharge test under a 200 Ω load, observing an approximately ∼20% decay in current over this period (Figure S6). Though surprisingly few studies demonstrate long-term (>1 h) cell testing under load,^9,11,17^ we note that the current decay observed under our 20 h discharge is comparable in magnitude to that observed in previous works over a 2 h discharge.^17,23,29,39,40^

To improve further upon this stable power output, we also probed the effect of two different electrode structures on the voltage–current density and power density–current density curves for our NaBr-supplemented hydrogel [Ni­(bpy)_3_]­Br_2_ electrolyte at Δ*T* = 20–60 K. Due to their higher surface area, superior ion diffusion through porous structures, and ability to enable more efficient charge transfer, carbon cloth electrodes were found to exhibit more than twice the current density and power density of graphite sheet electrodes, surpassing the typical power and current enhancements observed among the same electrodes in previous cells based on [Fe­(CN)_6_]^4–/3–^. Maximum normalized power (∼0.5 to 1 mW K^–2^ m^–2^) and relative Carnot efficiency (∼1 to 2%) plots for both electrode structures are shown in Figure 3e across Δ*T* ranges, demonstrating robust thermogalvanic performance stability over a wide thermal operating window. Additional experimental details are included in Figure S7.

Finally, Figure 3f summarizes the performance of the optimized [Ni­(bpy)_3_]^2+/3+^ cell, achieving simultaneously high voltage (∼200 mV), relative Carnot efficiency (η_r_ ∼ 2%), and functionality across a wide Δ*T* range (10–60 K). Compared with previously reported thermogalvanic redox cells, which we found to typically possess maximum outputs below 100 mV, relative Carnot efficiencies under 0.5%, and usable thermal windows less than 40 K, this system demonstrates exceptional potential for advanced thermoelectric heat conversion applications.

In conclusion, this study demonstrates that the [Ni­(bpy)_3_]^2+/3+^ redox couple offers a robust alternative to [Fe­(CN)_6_]^4–/3–^ for the design of aqueous thermogalvanic cells, exhibiting approximately double the Seebeck coefficient (∼2.86 mV/K), exceptionally high voltage (∼180 mV), and stable operation across a wide range of Δ*T* (up to 60 K). The self-sustaining [Ni­(bpy)_3_]^2+/3+^ redox cycle, driven by a large solvation entropy difference, ensures exceptional voltage stability and cyclability over many hours, and simple modifications to the electrolyte (gelation and additional charge carriers) further boost thermopower and power density. These findings suggest that [Ni­(bpy)_3_]^2+/3+^ may provide a promising redox platform for next-generation, high-thermopower thermogalvanic devices for low-grade waste heat recovery.

## Methods

### Materials

Tris­(2,2′-bipyridine)­nickel­(II) bromide (Ni­(bpy)_3_Br_2_, 95%) and polyvinyl alcohol (PVA, 87% hydrolyzed, 99%) were purchased from Ambeed. Sodium bromide (NaBr, ≥99%) was obtained from Strem Chemicals. Graphite electrodes (electrical resistivity of ∼10 μΩ·m) were commercial products sourced from Graphite Material Company Ltd. Commercially available unidirectional Hexcel IM7 Intermediate Modulus CFs were used as carbon cloth electrodes. Potassium ferrocyanide and potassium ferricyanide (98%) were obtained from BTC. All chemicals and materials were used as received without further purification. Deionized water (18.2 MΩ·cm, Milli-Q) was used as a solvent for the aqueous solutions.

### Cell Fabrication and Preparation

The prototype thermogalvanic cell was 3D-printed using resin, with a 10 mm × 10 mm × 35 mm liquid cell between two electrodes with an electrode–electrolyte contact area footprint of 1 cm^2^. We used graphite sheet electrodes in all experiments, except in Figure 3e,f, wherein we used electrodes prepared from commercial carbon cloth. These electrodes were prepared by immersion three times in acetone for 20 min each, which ensures removal of residual resin coating. The carbon cloth electrodes had an electrical resistivity of about 15 μΩ m, and the cell was sealed with Sil-Poxy rubber silicone adhesive. Two commercial Peltier cells (4 cm × 4 cm) maintained the desired temperature at the electrodes. Thermocouples, positioned between the electrodes and Peltier cells, ensured precise temperature control. Thermal insulation tape was applied to minimize heat loss. Copper tape and wire acted as collectors, providing low-resistance, and reliable connections for output measurements. Without a temperature difference (both electrodes at the same temperature), the average temperature was 296 K. When a temperature gradient was introduced, the cold electrode was held at 283 K, with key tests performed at hot-side temperatures of 293, 303, 313, 323, 333, and 343 K. We note that in preliminary investigations at 353 and 363 K, thermogalvanic output was yet retained but with performance deviating from the linear Seebeck behavior that describes the experiments reported herein. We also note that, given the aqueous nature of the electrolyte, experiments should not be conducted at temperatures higher than 373 K, for risk of boiling. In future work, alternative solvents with higher boiling points may be used to widen this temperature window of use.

To prepare the liquid electrolyte, aqueous solutions of tris­(2,2′-bipyridine)­nickel­(II) bromide ([Ni­(bpy)_3_]­Br_2_) were formulated at concentrations of 0.01, 0.02, 0.03, 0.04, and 0.05 M. Each solution was prepared by dissolving the appropriate mass of [Ni­(bpy)_3_]­Br_2_ in deionized water. The mixtures were heated to 50 °C on a hot plate and stirred at a low rate (approximately 100–150 rpm) for 12 h to ensure homogeneous dissolution and uniform distribution of the complex. To modulate the electrical conductivity, sodium bromide (NaBr) was added at concentrations of 0.1 and 0.5 M. These solutions were subsequently stirred at a low rate for an additional 12 h to fully integrate the NaBr with the [Ni­(bpy)_3_]­Br_2_ electrolyte, enhancing ionic conductivity by increasing the availability of mobile ions.

For the hydrogel electrolyte, a 10% (w/v) polyvinyl alcohol (PVA) solution was prepared by dissolving PVA in deionized water. The mixture was maintained at 80 °C for 48 h under gentle stirring to achieve a uniform, viscous solution free of aggregates. Similar to the liquid electrolyte, [Ni­(bpy)_3_]­Br_2_ was incorporated into the PVA solution at concentrations of 0.01, 0.02, 0.03, 0.04, and 0.05 M. These mixtures were stirred at 50 °C for 4 h to ensure thorough dispersion of the nickel complex within the polymer matrix. To enhance the electrical conductivity, NaBr was added at concentrations of 0.1 and 0.5 M, followed by additional stirring to promote uniform ion distribution.

To form the hydrogel’s cross-linked structure, the PVA-based samples underwent a freeze–thaw cycling process. The solutions were frozen at −20 °C for 12 h to induce physical cross-linking through the formation of crystalline PVA domains. After freezing, the samples were thawed at room temperature (approximately 25 °C) for 6–8 h. This freeze–thaw cycle was repeated four times to enhance the mechanical stability and cross-linking density of the hydrogel, resulting in a robust ion-conductive matrix suitable for electrochemical applications.

### Cell Characterization

Voltage and current were measured using a Keithley-2000 digital multimeter with data logged via MATLAB. A resistance board was incorporated into the circuit to monitor the working voltage and current during discharge. During charge–discharge cycling, the cell was charged at a constant temperature difference until the open-circuit voltage stabilized. Depending on the experiment, the cell was either short-circuited by setting the resistance to zero or connected to a closed circuit with a fixed resistance to mimic operational conditions.

## Supplementary Material



## Notes

Additional experimental and simulation details pertaining to characterization of thermal conductivity, thermopower, energy conversion efficiency, and relative Carnot efficiency, and the performance of all molecular dynamics simulations; Figures S1–S9 that include additional support for our experimental and simulation results, as referenced throughout the main text (PDF)
