# Supplementary material for: On a Continuous Aqueous Thermogalvanic Redox Agent with Anomalous Thermopower
Source: Nano Lett. 2025 Jul 24;25(31):11986–92. doi: 10.1021/acs.nanolett.5c02774 (PMC12333407; doi:10.1021/acs.nanolett.5c02774)
Supplement: Supplementary file 1 [file nl5c02774_si_001.pdf]

Supporting Information for

On a continuous aqueous thermogalvanic redox agent with anomalous thermopower

Ehsan Hosseini[a†^], Mohammad Zakertabrizi[a†], Mina Hosseini[a], Matthew. J. Powell-Palm[a,b,c ^]

[a]J. Mike Walker ’66 Dept. of Mechanical Engineering, Texas A&M University, College Station, TX 77803, USA

[b]Dept. of Materials Science and Engineering, Texas A&M University, College Station, TX 77803, USA

[c]Dept. of Biomedical Engineering, Texas A&M University, College Station, TX 77803, USA

[†]Equal contribution

[^]*To whom correspondence should be addressed:*

E.H. (ehsan.hosseini@tamu.edu)

M.J.P.P. (powellpalm@tamu.edu)

This Word document file includes:

Supplementary Note 1

Supplementary Figures S1 to S9

SUPPLEMENTARY NOTE 1: EXPERIMENTAL, THEORETICAL, AND COMPUTATIONAL PROCEDURES

Preparation of electrolyte

The ratios for potassium ferrocyanide ($K_4[Fe(CN)_6]$) and potassium ferricyanide ($K_3[Fe(CN)_6]$) in this study were adopted from Jin, Liyu, et al., and used as a benchmark to compare with our experimental setup[1].

Thermal conductivity

We employed the hotwire technique to measure the electrolyte's thermal conductivity. A detailed explanation of the method exists elsewhere, but a brief overview follows. A platinum (Pt) wire (7.5 cm long, 0.002794 cm diameter) was fixed inside a 10 cm × 5 cm × 5 cm container, with its ends soldered to copper wires. The container was filled with electrolyte liquid, and the wire served as both a heater and a thermometer based on its resistance. The copper wires were connected to a Keithley 2425 current source and a Keithley 2000 multimeter in a four-point configuration, both controlled via MATLAB using GPIB connections. A steady current (0.05 A) was applied for 0.9 seconds to measure voltage. The transient temperature rise ΔT was determined from the resistance change relative to the initial resistance, as described in equation (1).

$$\Delta T = \frac{\Delta R}{R_{ref}\beta_{ref}} \qquad (1)$$

The temperature coefficient of resistivity for the wire is βref = 3.729×10^{-3} K^{-1}, and R_{ref} represents the wire's resistance at 25 °C. The heat generation rate per unit length of the Pt wire (q) was calculated using equation (2).

$$q = \frac{I^2 R}{L} \qquad (2)$$

The thermal conductivity was calculated using equation (3), fitted to the linear portion of the plot on a logarithmic time scale. Here, L is the wire's length, R is the wire's resistance at the start of the measurement, and I is the constant current supplied by the source meter.

$$k = \frac{q/4\pi}{d(deltaT)/d(lnt)} \qquad (3)$$

The setup was validated by measuring the thermal conductivity of deionized (DI) water and ethylene glycol.

Theoretical methods

Thermopower is defined as the ratio of the induced electric field $-dT/dx$ to the applied temperature gradient dT/dx , as shown in equation (4).

$$S = \frac{-dV/dx}{dT/dx} = -\frac{dV}{dT} \qquad (4)$$

where S is the thermopower, V is the voltage, T is the temperature difference between the hot and the cold electrodes. However, this is only considering the voltage produced from the applied temperature difference. In our design, we deduct the voltage of the device in the ambient condition from the same device working under stable temperature difference to calculate the thermopower. *S* is also called *Seebeck* coefficient and is used interchangeably with thermopower. In this work, we used the isolated voltage as the numerator. The isolated voltage is the difference between the potential difference measured in the cell working ambient conditions and under controlled temperature difference. This value contains all contributing factors that become active under a heat gradient, namely the thermodiffusive and the redox factors.

The energy conversion efficiency and Carnot relative efficiency

The energy conversion efficiency is defined as the ratio of input heat and maximum output power as in equation (5).

$$\eta = \frac{P_{max}}{P_{heat}} = \frac{P_{max}}{\left(\frac{\kappa \Delta T}{d}\right)} \qquad (5)$$

Where P_{max} is maximum power, d is the distance between two electrodes, κ is the thermal conductivity and ΔT is temperature difference between the two electrodes.

The maximum power is calculated according to equation (6). We determined the thermogalvanic power output by deducting the isothermal maximum output power ($\Delta T = 0$ K) from the maximum output power from the cell operating under a temperature gradient ($\Delta T = 20$ K, $\Delta T = 40$ K, $\Delta T = 60$ K).

$$P_{max} = \frac{(V_{oc} I_{sc})_{\Delta T}}{4A} \qquad (6)$$

Where A is the single surface area of the electrode, V_{oc} is the close-circuit voltage and I_{sc} is the short circuit voltage.

Carnot-relative efficiency is the energy conversion efficiency over the relative temperature difference calculated in the following equation (7):

The Carnot-relative efficiency is defined as the energy conversion efficiency divided by the relative temperature difference, calculated using equation (7). Here, A represents the surface area of a single electrode, Voc is the open-circuit voltage, and Isc is the short-circuit current.

$$\eta_r = \frac{\eta}{\left(\frac{\Delta T}{T_{hot}}\right)} = \frac{P_{max} d T_{hot}}{k(\Delta T)^2} \qquad (7)$$

The maximum power output (Pmax) is determined using equation (6), where d is the distance between the electrodes, k is the thermal conductivity, and ΔT is the temperature difference between the electrodes.

Molecular Dynamics Simulations

In this work, we conducted molecular dynamics (MD) simulations using the Large-scale Atomic/Molecular Massively Parallel Simulator (LAMMPS) and Materials Studio to examine cooperative interactions within the electrolyte solution. Two distinct models were developed in a 3D periodic simulation box. The first model incorporated K^+ ions, $[Fe(CN)_6]^{4-/3-}$ complexes, and H_2O molecules, while the second included Br^+ ions, $[Ni(bpy)_3]^{2+/3+}$ complexes, and H_2O molecules. These compositions were chosen to ensure the system remained electrically neutral, a key requirement for realistic physical simulations. The simulation box was configured with dimensions of 64 Å × 64 Å × 32 Å to reflect the experimental conditions.

We constructed the initial electrolyte model using a carefully selected forcefield to accurately describe interactions among all species. Particular emphasis was placed on parameterizing the $[Fe(CN)_6]^{4-/3-}$ and $[Ni(bpy)_3]^{2+/3+}$ complexes, given their intricate coordination structures involving transition metals and cyanide or bipyridine ligands. Interaction parameters were adapted from the work of Prampolini, Giacomo, et al. [2]. We utilized parameters from Prampolini, Giacomo, et al., which provided surface charge details and interaction potentials tailored for our molecular dynamics (MD) simulations, along with atomic charges for the $[Fe(CN)_6]^{4-/3-}$ and $[Ni(bpy)_3]^{2+/3+}$ complexes derived from density functional theory (DFT). Other species, including K^+, Br^+, and H_2O, were parameterized using the Universal Forcefield (UFF) [3, 4] To ensure consistency across all species in the system, we adopted parameters from the literature, aligning interaction potentials, bond lengths, and angles with experimentally validated values.

Prior to starting the production simulation, ensuring the system reached a stable equilibrium was essential. This was accomplished using the SMART method, a multi-step minimization algorithm integrating three approaches: steepest descent, conjugate gradient, and Newton-Raphson. Initially, the steepest descent method addressed large overlaps or instabilities by gradually relaxing the structure along the steepest energy surface slope. Next, the conjugate gradient method refined the system by optimizing energy based on atomic forces, offering a more efficient minimization than steepest descent. Finally, the Newton-Raphson method provided high-precision adjustments in the concluding phase, ensuring the system was fully relaxed for the dynamics phase.

The system was equilibrated in the NPT ensemble for 10 nanoseconds to stabilize both temperature and pressure. The target temperature was set to 298 K, and the pressure was maintained at 1 atmosphere using a Nosé-Hoover thermostat and barostat [5]. This equilibration step allowed the simulation box volume to adjust naturally, ensuring an accurate representation of the electrolyte solution's density. Following NPT ensemble equilibration, the system was switched to the NVT ensemble for an additional 5 nanoseconds. In the NVT ensemble, the volume was held constant while maintaining the temperature at 298 K using a Nosé-Hoover thermostat. This fixed-volume environment was ideal for analyzing system dynamics without volume fluctuations, facilitating calculations such as diffusion analysis.

To investigate the structural organization of ions and molecules in the electrolyte, radial distribution functions (RDFs) were computed. RDFs describe the probability of finding pairs of

atoms at specific distances, normalized by the system's average density. Our analysis focused on RDFs for key species, including $[Fe(CN)_6]^{4-/3-}$ and $[Ni(bpy)_3]^{2+/3+}$, as well as other relevant ion pairs.

We determined the formation energy and observed bond-breaking phenomena. The formation energy was calculated using the equation outlined in equation (8):

$$E_{formation} = E_{complex} - \sum_{i} E_{isolated\,species} \tag{8}$$

$E_{complex}$ represents the total energy of the optimized $[Ni(bpy)_3]^{2+/3+}$ or $[Fe(CN)_6]^{4-/3-}$ system with H_2O, while $E_{isolated}$ species corresponds to the energies of the isolated H_2O and $[Ni(bpy)_3]$ or $[Fe(CN)_6]$ species. This formula provides an accurate method to evaluate the relative stability of various configurations.

All simulations were performed in triplicate, and for calculation of formation energies, at least five molecules per ion were sampled. Uncertainty in formation energy calculations is reported as the standard deviation across all sampled molecules for each condition (i.e. at least n = 15 molecules each).

SUPPLEMENTARY FIGURES

Fig S1 displays the energy density (kJ/m²) as a function of resistance (Ω) on a logarithmic scale for liquid and gel electrolyte phases, revealing distinct performance characteristics. Both phases exhibit a bell-shaped curve.

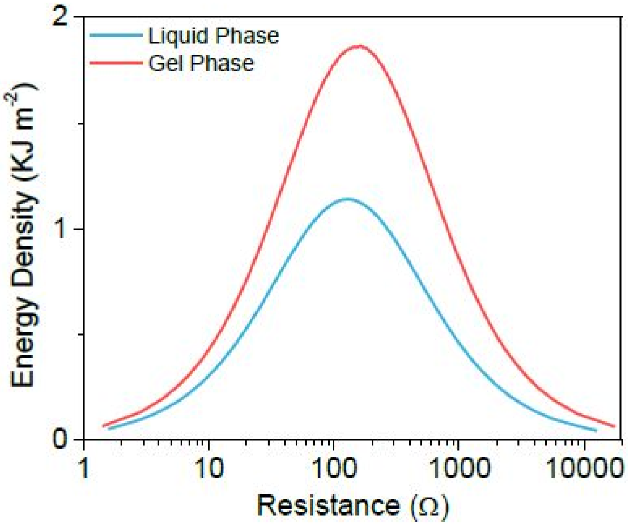

Figure S1. Energy density (kJ/m²) versus resistance (Ω) for liquid and gel electrolyte phases.

Fig S2 compares the thermal power density (W/m², left y-axis) and thermal energy density (kJ/m², right y-axis) of liquid and gel electrolytes at applied temperature differences of 20 K, 40 K, and 60 K. At 20 K.

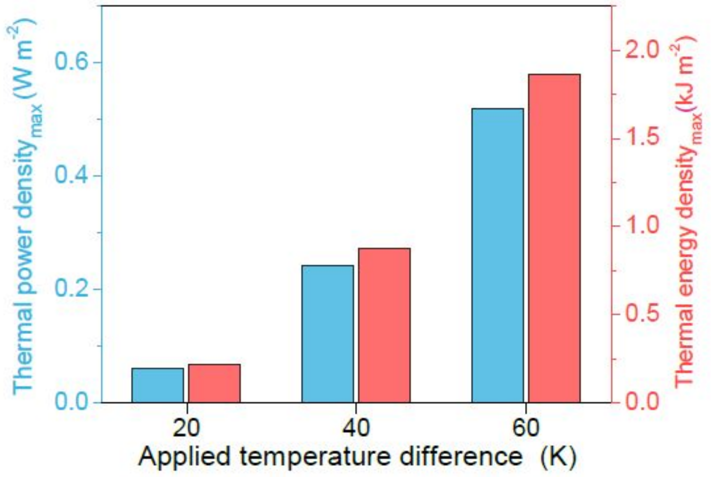

Figure S2. Energy density (kJ/m²) versus resistance (Ω) for liquid and gel electrolyte phases.

Fig S3 illustrates the normalized maximum power (Pmax/ΔT^2, in mW/m²·K²) of a gel electrolyte at applied temperature differences of 20 K, 40 K, and 60 K, with all bars consistently showing a value of approximately 0.15 mW/m²·K² across the range.

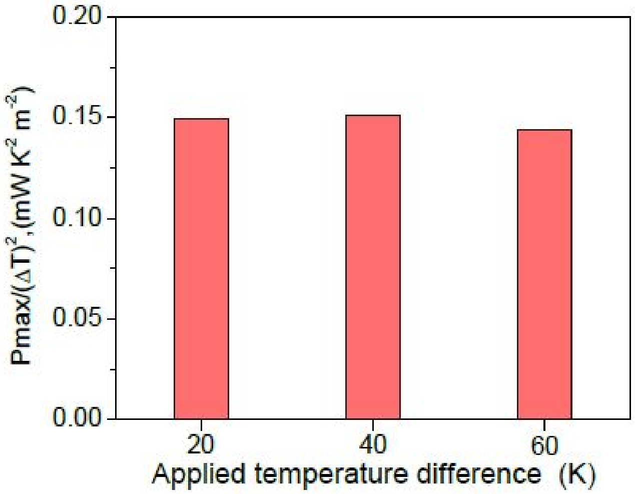

Figure S3. Normalized maximum power (Pmax/ΔT^2, mW/m²·K²) of a gel electrolyte at temperature differences of 20 K, 40 K, and 60 K.

Fig S4 displays the voltage of the $[Ni(bpy)_3]^{2+/3+}$ cell after 20 days of operation under a temperature gradient of ΔT = 60 K. This cell is the same as the one used in Figure 1f, and its performance was retested after 20 days. The results show that the cell can fully recover to its maximum thermopower.

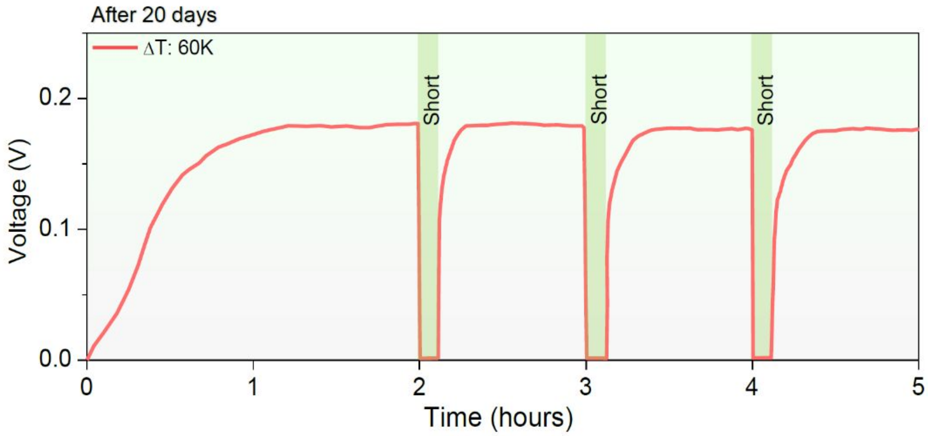

Figure S4. Long-Term Stability and Thermopower Recovery of $[Ni(bpy)_3]^{2+/3+}$ Cell After 20 Days at ΔT = 60 K

Fig S5 compares the electrical conductivity (S/m) of a Liquid electrolyte at two sodium bromide (NaBr) concentrations: 0.0 M and 0.5 M, showing a significant increase from approximately 0.65 S/m to 5.3 S/m as the NaBr concentration rises. This enhancement in conductivity is likely due to the increased availability of mobile ions (Br^- and Na^+) at the higher concentration, which facilitates greater charge transport within the Liquid or gel, a critical factor for improving the performance of thermogalvanic cells.

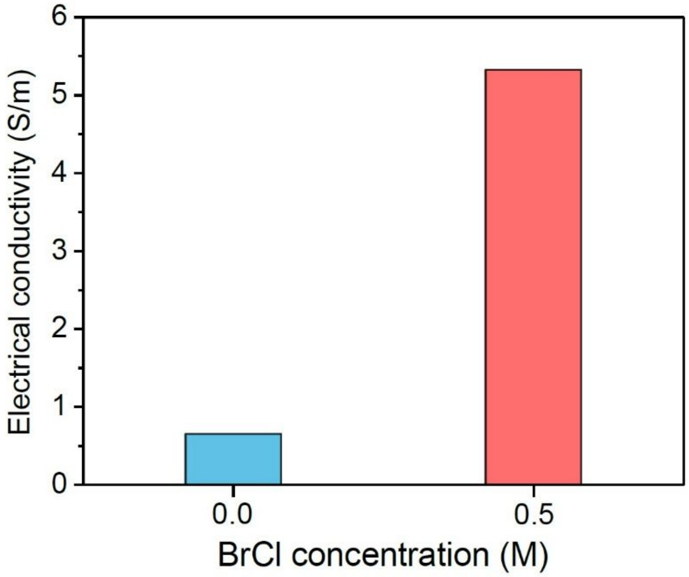

Figure S5. Electrical conductivity (S/m) of a gel electrolyte at NaBr concentrations of 0.0 M and 0.5 M.

Figure S6 shows the voltage and current output stability of a $[Ni(bpy)_3]Br_2$ at 0.05 M with NaBr at 0.5 M solution over 20 hours under a ΔT of 60 K (cold side 10 K, hot side 70 K), measured at a 200 Ω load. Focusing on continuous power, the voltage starts at approximately 155 mV and declines steadily to around 130 mV, while the current decreases from about 0.8 mA to 0.64 mA by the 20-hour mark.

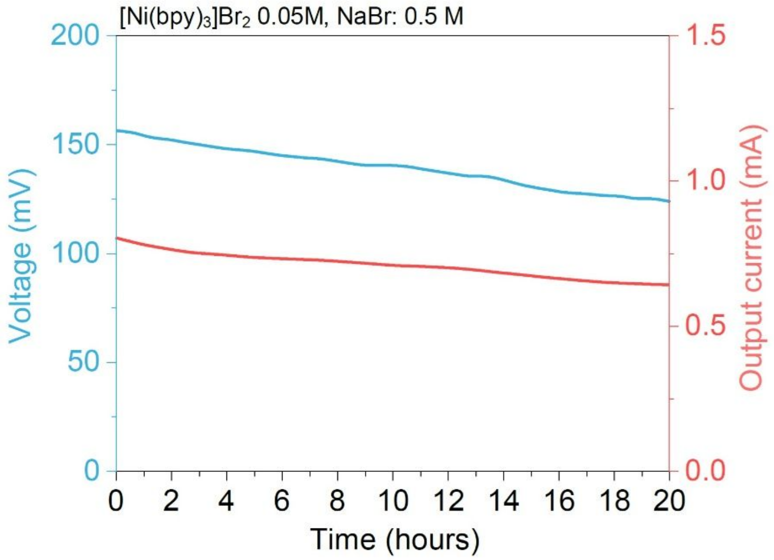

Figure S6. Voltage and current output stability of $[Ni(bpy)_3]Br_2$ at 0.05 M with NaBr at 0.5 M under ΔT of 60 k over 20 hours, with Voltage and current output measured at 200 Ω load.

Fig S7 illustrates the thermal conductivity (W/m·K) of a gel electrolyte containing 0.05 M $[Ni(bpy)_3]Br_2$ and 0.5 M NaBr at temperature differences of 20 K, 40 K, and 60 K, with values ranging from approximately 0.543 W/m·K at 20 K to 0.606 W/m·K at 60 K.

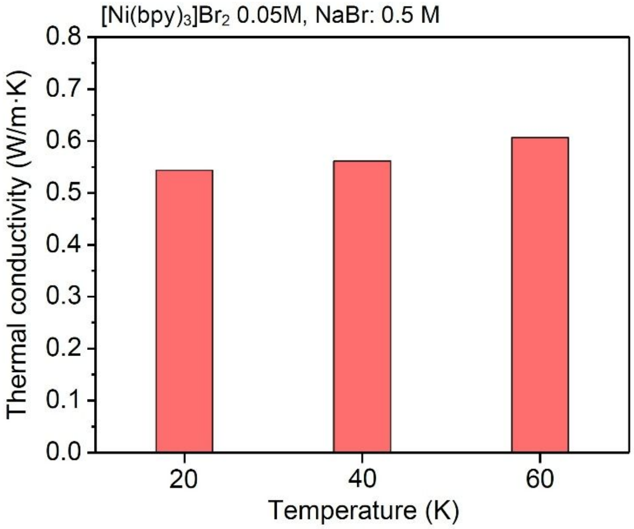

Figure S7. Thermal conductivity (W/m·K) of a gel electrolyte with 0.05 M $[Ni(bpy)_3]Br_2$ and 0.5 M NaBr at temperature differences of 20 K, 40 K, and 60 K.

Fig S8 depicts a molecular dynamics simulation snapshot of an electrolyte system within a 3D periodic box, showing water molecules and $[Ni(bpy)_3]^{2+}$ complexes. This model was developed for $[Ni(bpy)_3]^{3+}$, $[Fe(CN)_6]^{4-}$, and $[Fe(CN)_6]^{3-}$ complexes.

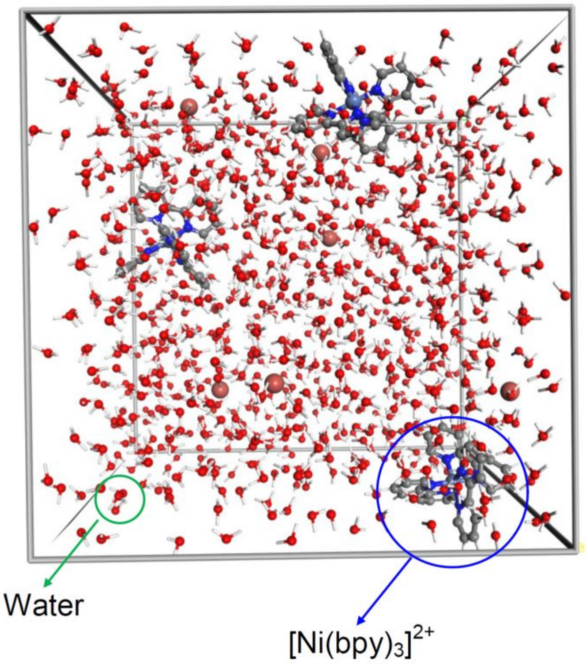

Figure S8. Molecular dynamics simulation snapshot showing $[Ni(bpy)_3]^{2+}$ complexes in a water-based electrolyte (red and white.

Supplementary References

1. Jin, L., et al., *Redox-active quasi-solid-state electrolytes for thermal energy harvesting.* ACS Energy Letters, 2016. **1**(4): p. 654-658.
2. Prampolini, G., et al., *Structure and Dynamics of Ferrocyanide and Ferricyanide Anions in Water and Heavy Water: An Insight by MD Simulations and 2D IR Spectroscopy.* The Journal of Physical Chemistry B, 2014. **118**(51): p. 14899-14912.
3. Rappe, A., K. Colwell, and C. Casewit, *Application of a universal force field to metal complexes.* Inorganic Chemistry, 1993. **32**(16): p. 3438-3450.
4. Rappé, A.K., et al., *UFF, a full periodic table force field for molecular mechanics and molecular dynamics simulations.* Journal of the American chemical society, 1992. **114**(25): p. 10024-10035.
5. Martyna, G.J., M.L. Klein, and M. Tuckerman, *Nosé–Hoover chains: The canonical ensemble via continuous dynamics.* The Journal of chemical physics, 1992. **97**(4): p. 2635-2643.
